# Effect of advanced chelate technology based trace minerals on growth performance, mineral digestibility, tibia characteristics, and antioxidant status in broiler chickens

**DOI:** 10.1186/s12986-020-00520-5

**Published:** 2020-10-29

**Authors:** Hossein Ali Ghasemi, Iman Hajkhodadadi, Maryam Hafizi, Kamran Taherpour, Mohammad Hassan Nazaran

**Affiliations:** 1 Department of Animal Science, Faculty of Agriculture and Natural Resources, Arak University, Arāk, 38156-8-8349 Iran; 2 Department of Research and Development, Sodour Ahrar Shargh Company, Tehran, Iran; 3 Department of Animal Science, Faculty of Agriculture, Ilam University, Ilam, Iran

**Keywords:** Broilers chickens, Chelate technology, Mineral absorption, Tibia mineralization, Trace elements

## Abstract

**Background:**

Compared to the corresponding source of inorganic trace minerals (TM), chelated supplements are characterized by better physical heterogeneity and chemical stability and appear to be better absorbed in the gut due to possibly decreased interaction with other feed components.

**Methods:**

This study was designed in broiler chickens to determine the effects of replacing inorganic trace minerals (TM) with an advanced chelate technology based supplement (Bonzachicken) on growth performance, mineral digestibility, tibia bone quality, and antioxidant status. A total of 625 male 1-day-old broiler chickens were allocated to 25 pens and assigned to 5 dietary treatments in a completely randomized design. Chelated TM (CTM) supplement was compared at 3 levels to no TM (NTM) or inorganic TM. A corn–soy-based control diet was supplemented with inorganic TM at the commercially recommended levels (ITM), i.e., iron, zinc, manganese, copper, selenium, iodine, and chromium at 80, 92, 100, 16, 0.3, 1.2, and 0.1 mg/kg, respectively, and varying concentration of CTM, i.e., match to 25, 50, and 100% of the ITM (diets CTM25, CTM50, and CTM100, respectively).

**Results:**

Diets CTM50 and CTM100 increased average daily gain (ADG), European performance index (EPI), and tibia length compared to the NTM diet (*P* < 0.05). Broilers fed the CTM100 diet had lowest overall FCR and serum malondialdehyde level and highest EPI, tibia ash, zinc, manganese, and copper contents, and serum total antioxidant capacity (*P* < 0.05). The apparent ileal digestibilities of phosphorus and zinc were lower in the ITM group compared with the CTM25 and CTM50 groups (*P* < 0.05). Broiler chickens fed any of the diets, except diet CTM25, exhibited higher serum glutathione peroxidase and superoxide dismutase activities than those fed the NTM diet, where the best glutathione peroxidase activity was found for CTM100 treatment (*P* < 0.05).

**Conclusions:**

These results indicate that while CTM supplementation to 25 and 50% of the commercially recommended levels could support growth performance, bone mineralization, and antioxidant status, a totally replacing ITM by equivalent levels of CTM could also improve performance index and glutathione peroxidase activity of broiler chickens under the conditions of this study.

## Background

Trace minerals (TM) play vital roles in the growth and development of broiler chickens, not only in the formation of bone tissues but also in the health maintenance [1, 2]. Essential TM, including co-factors of antioxidant enzymatic systems, e.g., selenium (Se) as a component of glutathione peroxidase (GSH-Px), zinc (Zn), copper (Cu), and manganese (Mn) that activate superoxide dismutase (SOD), and iron (Fe) as part of catalase (CAT), are commonly provided in inorganic forms in poultry diets to meet the birds' nutritional needs [3, 4]. However, such inorganic forms tend to readily dissociate from inorganic salts when exposed to acidic pH in the upper section of the gastrointestinal tract (GIT), likely resulting in an increased incidence of antagonism with other dietary constituents in the GIT. This could reduce their availability for absorption and, consequently, increase their excretion into soil and water that contributes to the potential environmental pollution [5, 6].

Organic TM have been increasingly used in broiler diets due to their greater bioavailability compared with inorganic mineral salts, such as sulfates, oxides, carbonates, and phosphates [7–9]. Organic minerals contain a variety of compounds in the form of amino acid chelates or proteinates [2] and recently organic acid chelates [10]. Currently, the preference of TM sources for poultry feed is based on their cost–benefit ratio, which leads nutritionists to include the least expensive source in diet formulation. However, some previous studies have demonstrated that, when using chelated TM (CTM), dietary TM concentrations can be decreased because of their high bioavailability [11]. Even though CTM supplementation to broiler feed is not directly related to improved productive performance [6], a single or combination of organic minerals in poultry feeds has been reported to exert multiple positive effects. For instance, organic TM supplementation resulted in decreased fecal mineral excretion [9, 12], improved bone mineralization and bone size traits [13], higher uric acid and lower blood malondialdehyde (MDA) concentration [1], and improved SOD and GSH-Px activities [14, 15]. However, the use of several organic TM, including Fe, Cu, Mn, Zn, Se, iodine (I), and chromium (Cr) synchronously replacing inorganic TM, and what kind of impact they will have on the growth rate and nutritional responses of commercial broiler chickens needs further investigation.

Advanced Chelate Compounds technology is a new technology that can design and synthesize efficient structures in different fields of science including medicine, agriculture, livestock, and poultry [16, 17]. Based on this technology, several organic acids are polymerized under a controlled condition, and during the polymerization, mineral elements are bonded on the basis of their affinity to the specific organic acids used as chelating agents. To date, several forms of chelated mineral supplements have been developed for animal and poultry diets, and their efficacy has been confirmed in various studies [10, 15, 18]. On the basis of advanced chelate technology, the Bonzachicken TM supplement has been precisely synthesized to cover all recommended TM requirements according to broiler nutrition guide [19].

At present, data on the efficiency of the advanced chelate technology based TM in broiler chicken production are limited. Therefore, the objective of the current study was, therefore, to evaluate the effects of advanced chelate technology based TM (Bonzachicken), at equivalent or low inclusion levels completely replacing inorganic TM, on growth performance, mineral digestibility, tibia characteristics, and antioxidant status of commercial broiler chickens.

## Methods

### Preparation of chelated TM

Bonzachicken is a supplement manufactured on the basis of self-assembly method and according to the patent US8288587B2. The structure of this supplement is highly stable because of the shear-like enclosure of central ions by polymerized chelating agents. The stability of this chelated structure at various pH levels is presented in Table 1. As can be seen, there is little variation in the ppm of different elements at alkaline and acidic pH levels as compared to total, which reveals the stability of the chelated structure of Bonzachicken and its resistance to environmental pH alterations.

**Table 1:** Quantities of trace minerals present in Bonzachicken product

	Iron (ppm)	Zinc (ppm)	Copper (ppm)	Manganese (ppm)	Selenium (ppm)	Chromium (ppm)	Iodine (ppm)
Analytical method	AA	AA	AA	AA	Graphite furnace AA	AA	Calorimetry
Total	40,500	45,960	8200	50,503	158.0	56.0	612.0
pH = 4	40,405	46,000	8110	50,113	147.6	49.5	617.4
pH = 9	40,300	45,870	7943	49,802	160.0	50.0	609.8

*AA* Atomic absorption

### Broiler chickens and dietary treatments

A total of 625 male broiler chickens (Ross 308) at 0 day old (44 ± 1.15 g) were allocated to 5 dietary treatments, in a completely randomized design. Each of the 5 treatments had 5 replicates and 25 birds per replicate. The composition of starter (day 0–10), grower (day 10–24), and finisher (day 24–42) diets is given in Table 2. Experimental treatments were: (1) no TM (NTM), (2) commercially recommended levels of inorganic TM (ITM; 80 mg Fe as ferrous sulfate, 92 mg Zn as zinc sulphate, 100 mg Mn as manganese sulfate, 16 mg Cu as copper sulfate, 0.3 mg Se as sodium selenite, 1.2 mg I as potassium iodide, 0.1 mg Cr as potassium dichromate, per kilogram of diet); (3) very low CTM (CTM25; match to 25% of the ITM); (4) Low CTM (CTM50; match to 50% of the ITM); (5) high CTM (CTM100; equivalent to ITM). For the ITM100 treatment, the basal diet supplemented with 2.5 g/kg inorganic TM premix. For the CTM25, CTM50, and CTM100 treatments, the basal diet supplemented with 0.5, 1, and 2 g/kg of Bonzachicken supplement, respectively, to meet the defined TM levels for each treatment. The TM premixes were added in place of the building sand that is included in the NTM diet as inert filler. Dietary treatments varied in TM composition are shown in Table 3. Finisher diets were contained 0.5% titanium dioxide (TiO_2_) as an indigestible marker to estimate the apparent mineral digestibility in the ileum.

**Table 2:** Ingredient composition and calculated nutrient contents of basal diets (as-fed basis)

Item	Starter (day 0–10)	Grower (day 10–24)	Finisher (day 24–42)
Ingredients (%)			
Corn	55.56	57.86	61.04
Soybean meal, 44%	32.12	30.11	26.45
Corn gluten meal, 60%	5.28	4.22	3.24
Soybean oil	2.20	3.50	4.80
Dicalcium phosphate	1.95	1.71	1.50
Limestone	1.16	1.07	1.00
Salt (NaCl)	0.22	0.23	0.20
Sodium bicarbonate	0.11	0.10	0.14
Vitamin premix^1^	0.25	0.25	0.25
Trace mineral premix^2^	0–0.25	0–0.25	0–0.25
dl-Methionine	0.26	0.23	0.22
l-Lysine HCl	0.43	0.32	0.28
l-Threonine	0.21	0.15	0.13
TiO_2_	–	–	0.50
Building sand	0–0.25	0–0.25	0–0.25
Total	100	100	100
Calculated nutritive value			
Metabolizable energy, kcal/kg	3000	3100	3200
Crude protein (%)	23.0	21.5	19.5
Calcium (%)	0.96	0.87	0.79
Nonphytate phosphorus (%)	0.48	0.44	0.40
Sodium (%)	0.16	0.16	0.16
Digestible lysine (%)	1.28	1.15	1.03
Digestible methionine (%)	0.62	0.55	0.51
Digestible methionine + cysteine (%)	0.95	0.87	0.80
Digestible threonine (%)	0.86	0.77	0.69
DEB^3^, mEq/kg	250	240	230
Analysed values^4^			
Crude fat (%)	4.70 ± 0.09	6.10 ± 0.10	7.36 ± 0.12
Crude fiber (%)	3.64 ± 0.06	3.53 ± 0.08	3.41 ± 0.05
TiO_2_^4^ (%)	–	–	0.49 ± 0.01
Magnesium (%)	0.18 ± 0.007	0.17 ± 0.010	0.16 ± 0.009
Potassium (%)	0.93 ± 0.016	0.90 ± 0.012	0.85 ± 0.019
Sodium (%)	0.15 ± 0.006	0.16 ± 0.011	0.15 ± 0.008

^1^Supplied per kg diet: 18 mg retinol, 4 mg cholecalciferol, 36 mg a-tocopherol acetate, 2 mg vitamin K_3_, 1.75 mg vitamin B_1_, 6.6 mg vitamin B_2_, 9.8 mg niacin, 29.65 mg pantothenic acid, 2.94 mg vitamin B_6_, 1 mg folic acid, 0.015 mg vitamin B_12_, 0.1 mg biotin, 250 mg choline chloride and 1 mg ethoxyquin

^2^The trace mineral (TM) supplementation were referred to our experimental design. The TM premixes were added in place of the building sand that is used as inert filler to adjust the formulation

^3^DEB (dietary electrolyte balance) = (Na^+^, mEq/kg + K^+^, mEq/kg) − CL^−^, mEq/kg

^4^Mean and standard deviation of 3 samples/diet (without any supplement)

**Table 3:** Supplemental level of trace minerals and analyzed contents of protein and minerals in experimental diets

Item	Experimental treatments^1^				
	NTM	INORG100	CTM25	CTM50	CTM100
Supplemental level					
Iron (mg/kg)	0	80	20	40	80
Zinc (mg/kg)	0	92	23	46	92
Manganese (mg/kg)	0	100	25	50	100
Copper (mg/kg)	0	16	4	8	16
Selenium (mg/kg)	0	0.3	0.08	0.15	0.3
Iodine (mg/kg)	0	1.2	0.3	0.6	1.2
Chromium (mg/kg)	0	0.1	0.03	0.05	0.1
*Analyzed mineral content*					
Starter (day 0–10)					
Crude protein (g/kg)	227 ± 3.2	225 ± 2.1	226 ± 2.0	228 ± 2.7	227 ± 1.8
Calcium (g/kg)	9.7 ± 0.07	9.9 ± 0.12	9.8 ± 0.06	9.6 ± 0.14	9.7 ± 0.10
Total phosphorus (g/kg)	7.8 ± 0.08	7.6 ± 0.06	7.7 ± 0.08	7.6 ± 0.10	7.5 ± 0.09
Iron (mg/kg)	66 ± 0.62	148 ± 1.35	84 ± 1.12	108 ± 0.95	145 ± 1.43
Zinc (mg/kg)	33 ± 0.81	123 ± 1.40	55 ± 1.21	80 ± 0.72	127 ± 1.13
Manganese (mg/kg)	42 ± 0.41	143 ± 1.15	64 ± 0.80	95 ± 1.20	139 ± 2.02
Copper (mg/kg)	3.2 ± 0.08	19.7 ± 0.26	7.4 ± 0.14	11.3 ± 0.20	20.1 ± 0.39
Selenium (mg/kg)	0.15 ± 0.008	0.44 ± 0.018	0.23 ± 0.009	0.31 ± 0.013	0.47 ± 0.007
Iodine (mg/kg)	0.12 ± 0.006	1.23 ± 0.026	0.38 ± 0.017	0.70 ± 0.014	1.25 ± 0.020
Chromium (mg/kg)	ND^3^	0.096 ± 0.008	0.028 ± 0.003	0.054 ± 0.004	0.102 ± 0.006
Grower (day 10–24)					
Crude protein (g/kg)	213 ± 2.5	215 ± 2.0	211 ± 1.7	211 ± 1.5	213 ± 1.4
Calcium (g/kg)	8.9 ± 0.06	8.8 ± 0.09	9.1 ± 0.11	9.0 ± 0.08	8.9 ± 0.12
Total phosphorus (g/kg)	7.3 ± 0.05	7.0 ± 0.12	7.2 ± 0.07	6.9 ± 0.07	7.1 ± 0.11
Iron (mg/kg)	63 ± 0.55	143 ± 2.19	82 ± 1.07	105 ± 1.16	142 ± 1.45
Zinc (mg/kg)	33 ± 0.63	121 ± 1.13	55 ± 0.92	78 ± 1.03	118 ± 1.35
Manganese (mg/kg)	40 ± 0.35	145 ± 1.42	63 ± 1.22	92 ± 0.98	139 ± 1.18
Copper (mg/kg)	3.0 ± 0.06	19.3 ± 0.21	7.2 ± 0.14	11.5 ± 0.20	19.4 ± 0.22
Selenium (mg/kg)	0.15 ± 0.005	0.43 ± 0.012	0.21 ± 0.006	0.31 ± 0.012	0.46 ± 0.010
Iodine (mg/kg)	0.11 ± 0.008	1.25 ± 0.023	0.38 ± 0.009	0.69 ± 0.012	1.24 ± 0.014
Chromium (mg/kg)	ND	0.094 ± 0.006	0.026 ± 0.004	0.048 ± 0.005	0.099 ± 0.010
Finisher (day 24–42)					
Crude protein (g/kg)	194 ± 3.5	193 ± 3.0	193 ± 2.3	192 ± 1.9	193 ± 2.6
Calcium (g/kg)	8.2 ± 0.05	8.1 ± 0.15	8.2 ± 0.09	8.3 ± 0.07	8.0 ± 0.13
Total phosphorus (g/kg)	6.5 ± 0.07	6.3 ± 0.09	6.6 ± 0.12	6.5 ± 0.08	6.4 ± 0.14
Iron (mg/kg)	58 ± 0.64	143 ± 1.73	80 ± 1.25	103 ± 1.14	139 ± 1.51
Zinc (mg/kg)	30 ± 0.76	122 ± 1.55	54 ± 1.04	75 ± 0.86	118 ± 1.24
Manganese (mg/kg)	42 ± 0.50	141 ± 2.10	65 ± 1.10	95 ± 0.85	143 ± 1.45
Copper (mg/kg)	3.1 ± 0.08	19.7 ± 0.19	7.0 ± 0.14	11.8 ± 0.26	19.5 ± 0.32
Selenium (mg/kg)	0.14 ± 0.009	0.41 ± 0.014	0.22 ± 0.005	0.28 ± 0.010	0.43 ± 0.015
Iodine (mg/kg)	0.11 ± 0.010	1.27 ± 0.026	0.41 ± 0.012	0.70 ± 0.018	1.24 ± 0.024
Chromium (mg/kg)	ND	0.095 ± 0.006	0.027 ± 0.002	0.051 ± 0.005	0.104 ± 0.009

^1^NTM, no trace mineral supplementation; ITM, commercially recommended levels of inorganic trace mineral; CTM25, chelated trace mineral supplement (CTM) match to 25% of the ITM; CTM50, CTM match to 50% of the ITM; CTM100, CTM equivalent to ITM

^2^Mean and standard deviation of 3 samples/diet

^3^ND = nondetectable (< 2 µg/kg of diet)

All birds had free access to water (1 hanging drinker per pen) and feed (mash form). Drinking water was analyzed for minerals using an inductively coupled plasma—atomic emission spectroscopy (ICP-AES; Optima 7000 DV, Perkin Elmer, Waltham, MA) prior to the experiment. The concentrations of Ca, Mn, and Fe in the drinking water were 5.3, 0.06, and 0.03 mg/L, respectively, whereas the concentrations of P and other TM were undetectable in the water.

All experimental groups were housed in floor pens (length 175 cm × width 170 cm) using litter top dressed with 5 cm of clean wood shavings in an environmentally controlled house. The room temperature was set at 34 °C on the day of arrival, and then reduced by 0.40 °C per day until 24 °C, where it remained for the rest of the trial. The environmental relative humidity was maintained at 50–65% by periodical spraying the walkways with water and adjusting the humidifiers. The lighting program used was 24L∶0D from day 0 to 3 and 23L∶1D for the remainder of the experiment.

### Growth performance

Pen body weight (BW) and feed intake were recorded at placement, 10, 24, and 42 days of age for calculation of average daily gain (ADG) and average daily feed intake (ADFI) per bird for each replicate pen. Incidences of mortality were recorded daily in order to determine the mortality rate. With the body weight of any deceased or culled chickens included, total ADG and total ADFI for each pen were used for calculating the mortality-adjusted feed conversion ratio (FCR) during each feeding period. European performance index (EPI) per treatment was also estimated using the formula described by Ghasemi and Nari [20].

### Sample collection

On day 35, 2 chickens per replicate pen, each with a BW close to the average BW of each pen, were euthanized by cervical dislocation, and the digesta samples from the ileum (from Meckel's diverticulum up to 5 cm proximal to the ileocecal junction) were gently flushed into plastic tubes. The ileal digesta of two birds in a replicate were pooled, after which a representative sample was freeze-dried and ground to pass a 1 mm screen prior to chemical analysis.

At the end of the experiment (42 days), after overnight fasting, 2 birds per replicate pen were selected according to the average BW of the pen. Blood samples were collected from the brachial vein of each bird and then centrifuged (2500×*g*, 15 min) at 4 °C to obtain serum samples for blood chemical analysis. The above 2 birds from each cage were then euthanized by cervical dislocation followed blood sampling; both right and left tibia of each bird was excised and cleaned of adhering tissues. Right tibia was measured for tibia weight, length, and diameter, whereas left tibia kept frozen at − 20 °C until analysis for tibia ash, calcium, and phosphorous content.

### Chemical analysis and mineral digestibility

Duplicate samples of basal diets were analyzed for crude protein (N × 6.25; method 994.13), crude fat (method 920.39), and crude fiber (method 978.10) according to AOAC [21]. For mineral analysis, all diets and ileal digesta samples were milled to pass through 0.5 mm mesh sieve before analysis. Samples were subsequently analyzed for calcium (Ca), phosphorus (P), magnesium (Mg), potassium (K), sodium (Na), Fe, Zn, Mn, Cu, and TiO_2_. Mineral content in the diet and ileal digesta samples was determined using an ICP-AES [21] following digestion in concentrated HNO_3_. The total Se, I and Cr concentrations in the diets were also determined using an inductively coupled plasma mass spectrometry (ICP-MS) technique. Titanium dioxide analysis was done using the spectrophotometric method described by Short et al. [22]. The apparent ileal digestibility (day 35) of minerals was calculated using the following formula:

$${\text{Apparent}}\;{\text{ileal}}\;{\text{mineral}}\;{\text{digestibility}} = {1} - \left[ {\left( {{\text{mineral}}/{\text{TiO}}_{{2}} } \right)\;{\text{digesta}}/\left( {{\text{mineral}}/{\text{TiO}}_{{2}} } \right)\;{\text{diet}}} \right]$$

### Tibia bone parameters

The weight and length of the right tibia of each bird were measured. Tibia diameters were also measured at the widest and narrowest points using a digital caliper in mm to 2 decimals, and then averaged. The left tibia samples were crushed and defatted with petroleum ether for 24 h using Soxhlet apparatus, and dried in the oven at 100 °C for 24 h. Dried bone samples were then burned (24 h) into a muffle furnace preheated to 600 °C for determination of ash percentage (dry, fat-free basis). The ash from tibia samples was solubilized with a mixture of nitric and perchloric acid, and the contents of minerals were determined by the same methods as those applied for the samples from diet and excreta.

### Serum variables

Serum samples were used for the measurement of some metabolites, total antioxidant capacity (TAC), antioxidant enzyme activity, as well as MDA content. The serum concentrations of glucose, triglyceride, cholesterol, total protein, albumin, and uric acid were determined using a corresponding assay kit (Pars Azmoon Company, Tehran, Iran) and an automatic biochemical analyzer (Clima, Ral. Co, Spain). The activities of GSH-Px, SOD, and CAT were determined colorimetrically (enzymatically) and serum TAC and MDA contents were measured by assay kits for TAC (Randox Laboratories Ltd, Crumlin, UK), GSH-Px, SOD, CAT, MDA (Cayman Chemical Co., Ann Arbor, MI, USA).

### Statistical analysis

All the data were statistically analyzed as a completely randomized design using GLM procedures of SAS 9.4 (SAS Institute Inc., Cary NC) with a pen as an experimental unit. All percentage data were tested for normality by employing the UNIVARIATE procedure of SAS, then transformed to arcsine values before analysis if normality was not met. Mean separation was conducted by the Tukey's post-hoc analysis and Bonferroni correction with differences deemed significant at *P* < 0.05.

## Results

### Growth performance

The effects of dietary treatments on growth performance are presented in Table 4. There were no significant differences in ADFI and mortality rate of broilers during day 0–42 among different dietary treatments. The ADG of birds fed diet CTM100 was greater (*P* < 0.05) than that of birds fed the NTM diet during the starter rearing period. The CTM50 and CTM100 groups also exhibited higher ADG (*P* < 0.05) than NTM group during the overall experimental period. Although there were no treatment differences in FCR during each growth pahse, the CTM100 group had lower FCR (*P* < 0.05) than the NTM group in the overall experimental period (day 0–42).. When the broiler EPI for the entire study was calculated, the EPI of the ITM,CTM50 and CTM100 groups was greater (*P* < 0.05) than the NTM group. By comparison, broilers fed with the CTM100 diet had higher EPI (*P* < 0.05) than birds fed diets ITM and CTM25.

**Table 4:** Effect of experimental treatments on growth performance in broilers

Item	Experimental treatments^1^					SEM	*P* value
	NTM	ITM	CTM25	CTM50	CTM100		
Average daily gain (g/bird/day)							
0–10 days	15.18^b^	16.68^ab^	16.18^ab^	16.60^ab^	17.76^a^	0.42	0.007
10–24 days	54.83	57.44	56.41	58.17	60.28	1.36	0.103
24–42 days	80.84	86.39	84.11	87.17	88.87	2.06	0.097
0–42 days	56.54^b^	60.14^ab^	58.71^ab^	60.70^a^	62.41^a^	0.84	0.001
Average daily feed intake (g/bird/day)							
0–10 days	20.47	21.16	21.41	21.04	21.58	0.44	0.466
10–24 days	85.32	87.78	87.09	89.35	89.52	2.09	0.608
24–42 days	172.4	173.0	170.6	171.8	169.5	2.73	0.898
0–42 days	107.2	108.4	107.2	108.4	107.6	1.18	0.901
Feed conversion ratio							
0–10 days	1.35	1.27	1.32	1.28	1.22	0.03	0.061
10–24 days	1.56	1.53	1.54	1.54	1.49	0.05	0.910
24–42 days	2.14	2.00	2.05	1.97	1.91	0.07	0.060
0–42 days	1.90^a^	1.80^ab^	1.83^ab^	1.79^ab^	1.73^b^	0.04	0.036
Mortality rate (%)							
0–42 days	8.80	5.60	5.60	4.80	3.20	1.31	0.083
European performance index^2^							
0–42 days	277.3^c^	318.4^bc^	307.9^bc^	326.8^ab^	357.3^a^	10.65	< 0.001

Values are means of 5 pens per treatment combination with 25 male broiler chickens

^a,b,c^Values in the same row with different superscripts were significantly different (*P* < 0.05)

^1^NTM, no trace mineral supplementation; ITM, commercially recommended levels of inorganic trace mineral; CTM25, chelated trace mineral supplement (CTM) match to 25% of the ITM; CTM50, CTM match to 50% of the ITM; CTM100, CTM equivalent to ITM

^2^Calculated as: liveability (%) × live weight (kg) × 100/age (day) × (feed intake/body weight gain)

### Mineral digestibility

Apparent ileal digestibility (AID) values of macro and trace minerals on day 35 are shown in Table 5. The AID of Ca, Mg, K, Na, and Fe was not affected by dietary treatments (*P* > 0.05). In contrast, the AID of P was higher (*P* < 0.05) in broiler chickens fed diets CTM25 and CTM50, compared with those fed diet ITM. The AID of Zn in broiler chickens fed any of the diets, except diet CTM100, was significantly greater (*P* < 0.05) than in birds fed the ITM diet. Broiler chickens fed diet CTM25 also exhibited greater (*P* < 0.05) AID of Mn than those fed the ITM diet. In addition, the AID of Cu was higher (*P* < 0.05) in the NTM and CTM25 groups relative to the ITM group.

**Table 5:** Effect of experimental treatments on percent ileal absorption of minerals in broilers^1^ at day 35

Item	Experimental treatments^2^					SEM	*P* value
	NTM	ITM	CTM25	CTM50	CTM100		
Calcium	46.8	45.0	47.8	53.8	50.2	2.2	0.086
Phosphorus	59.4^ab^	55.8^b^	63.5^a^	62.9^a^	61.8^ab^	1.7	0.035
Magnesium	42.7	37.3	45.5	38.3	39.0	3.2	0.368
Potassium	79.1	79.5	80.4	78.2	80.2	1.3	0.732
Sodium	57.2	54.7	55.5	48.0	46.5	3.5	0.160
Iron	47.8	39.5	47.6	43.5	42.2	2.5	0.120
Zinc	29.4^a^	16.6^b^	34.3^a^	29.3^a^	27.9^ab^	3.7	0.040
Manganese	23.5^ab^	14.5^b^	30.9^a^	23.8^ab^	22.6^ab^	2.6	0.006
Copper	31.5^a^	21.4^b^	33.1^a^	27.2^ab^	23.7^ab^	2.7	0.027

^a,b,c^Values in the same row with different superscripts were significantly different (*P* < 0.05)

^1^Values are means of 5 pens per treatment combination with 2 male broiler chickens per pen selected for ileal digesta collection

^2^NTM, no trace mineral supplementation; ITM, commercially recommended levels of inorganic trace mineral; CTM25, chelated trace mineral supplement (CTM) match to 25% of the ITM; CTM50, CTM match to 50% of the ITM; CTM100, CTM equivalent to ITM

### Tibia characteristics

Tibia quality and tibia mineral contents on day 42 are detailed in Table 6. Dietary treatments did not affect tibia bone weight and tibia Ca, Mg, K, and Na concentrations (*P* > 0.05). However, tibia length of broiler chickens fed diets CTM50 and CTM100 was higher (*P* < 0.05) than that of birds fed the NTM diet. Moreover, broiler chickens fed with the CTM50 and CTM100 diets had increased tibia phosphorus content (*P* < 0.05) than those fed with the ITM diet. The tibia ash and Cu contents in broiler chickens fed diet CTM100 were higher than (*P* < 0.05) in birds fed the NTM diet. Diet CTM100 also increased tibia Zn concentration (*P* < 0.05) compared with the NTM and CTM25 diets. The tibia Mn content in the broiler chickens fed any of the diets was greater (*P* < 0.05) than those in birds fed the NTM diet, where birds fed the CTM100 diet had the highest tibia Mn content. By comparison, tibia Mn content in the ITM and CTM50 groups was also greater (*P* < 0.05) than that of NTM and CTM 25 groups.

**Table 6:** Effect of experimental treatments on tibia quality and tibial mineral content in broilers^1^ at day 42

Item	Experimental treatments^2^					SEM	*P* value
	NTM	ITM	CTM25	CTM50	CTM100		
Tibia length (cm)	9.48^b^	10.14^ab^	10.03^ab^	10.38^a^	10.29^a^	0.19	0.031
Tibia weight (g)	13.89	15.35	14.85	15.40	15.91	0.48	0.070
Ash (%)	51.12^b^	52.03^ab^	53.19^ab^	55.11^ab^	55.90^a^	1.08	0.026
Calcium (g/kg)	165.4	172.2	169.8	176.6	175.4	3.32	0.161
Phosphorus (g/kg)	95.36^ab^	92.27^b^	96.65^ab^	97.42^a^	98.33^a^	1.06	0.006
Magnesium (g/kg)	4.03	4.08	4.11	4.14	4.06	0.20	0.995
Potassium (g/kg)	2.02	1.99	2.01	2.08	2.14	0.10	0.810
Sodium (g/kg)	4.62	4.44	4.88	4.52	4.82	0.18	0.396
Iron (mg/kg)	128.4	131.8	133.6	135.0	133.3	2.77	0.547
Zinc (mg/kg)	155.8^b^	170.5^ab^	157.9^b^	165.2^ab^	179.0^a^	3.53	0.001
Manganese (mg/kg)	3.30^d^	4.45^ab^	3.82^c^	4.40^b^	4.87^a^	0.10	< 0.001
Copper (mg/kg)	0.68^b^	0.83^ab^	0.78^ab^	0.82^ab^	0.87^a^	0.03	0.011

^a,b,c,d^Values in the same row with different superscripts were significantly different (*P* < 0.05)

^1^Values are means of 5 pens per treatment combination with 2 male broiler chickens per pen selected for tibia collection

^2^NTM, no trace mineral supplementation; ITM, commercially recommended levels of inorganic trace mineral; CTM25, chelated trace mineral supplement (CTM) match to 25% of the ITM; CTM50, CTM match to 50% of the ITM; CTM100, CTM equivalent to ITM

### Blood metabolites and antioxidant status

As shown in Table 7, at 42 days of age, there were no differences in serum glucose, triglyceride, cholesterol, protein, and albumin concentrations among treatment groups (*P* > 0.05). In contrast, uric acid concentration in broiler chickens fed diet CTM100 was higher (*P* < 0.05) than those fed diets NTM and ITM.

**Table 7:** Effect of experimental treatments on serum metabolites and antioxidant profile of broilers^1^ at day 42

Item	Experimental treatments^2^					SEM	*P* value
	NTM	ITM	CTM25	CTM50	CTM100		
Glucose (mg/dL)	258.8	242.9	263.1	257.2	259.5	16.10	0.915
Triglyceride (mg/dL)	63.96	59.64	59.58	60.16	65.04	7.62	0.975
Cholesterol (mg/dL)	114.4	103.4	109.6	103.6	101.6	6.23	0.579
Protein (g/dL)	4.56	4.38	3.98	4.68	4.24	0.18	0.100
Albumin (g/dL)	2.50	2.26	2.36	2.50	2.04	0.28	0.759
Uric acid (mg/dL)	7.40^b^	7.56^b^	7.85^ab^	8.60^ab^	9.33^a^	0.39	0.012
*Antioxidant status*							
Total antioxidant capacity (U/mL)	3.22^b^	4.46^ab^	4.13^ab^	4.31^ab^	5.04^a^	0.34	0.017
Glutathione peroxidase (U/mL)	1132^c^	1481^b^	1412^bc^	1516^ab^	1812^a^	70.30	< 0.001
Superoxide dismutase (U/mL)	185.3^b^	244.7^a^	220.6^ab^	238.9^a^	254.3^a^	10.42	0.001
Catalase (U/mL)	1.80	2.40	1.96	2.22	2.15	0.18	0.198
Malondialdehyde (nmol/mL)	2.88^a^	2.22^ab^	2.26^ab^	2.16^ab^	1.65^b^	0.19	0.004

^a,b,c^Values in the same row with different superscripts were significantly different (*P* < 0.05)

^1^Values are means of 5 pens per treatment combination with 2 male broiler chickens per pen selected for blood collection

^2^NTM, no trace mineral supplementation; ITM, commercially recommended levels of inorganic trace mineral; CTM25, chelated trace mineral supplement (CTM) match to 25% of the ITM; CTM50, CTM match to 50% of the ITM; CTM100, CTM equivalent to ITM

Diet CTM100 increased serum TAC concentration (*P* < 0.05) compared to the NTM diet (Table 7). At 42 days, the activity of GSH-Px in the ITM, CTM50, and CTM100 groups was also higher (*P* < 0.05) than that in the NTM group, where birds fed the CTM100 diet had the highest GSH-Px activity. The SOD activity of birds fed any of the diets, except diet CTM25, was higher (*P* < 0.05) than in the NTM group.The serum MDA concentration was also decreased (*P* < 0.05) in the CTM100 group compared with the NTM group.

## Discussion

In this study, the deficiency of TM (Zn, Fe, Mn, Cu, Se, I, and Cr) in the NTM diet strongly depressed overall growth performance of broilers, which was more noticeable during the early growth period. This normally can be linked to higher rates of growth, bone development, and metabolism during this period [7]. Based on the results of the current study, the CTM25, CTM50, and ITM treatment groups showed no significant differences on growth performance. The results also revealed that although there was no difference between CTM50 and CTM100 groups on production performances, only the CTM100 diet increased EPI during the overall growth period compared with equal levels of TM from ITM treatment, which indicated that the complete substitution of inorganic TM sources by CTM could be as an alternative way to improve productive performance. The improvements in growth performance have been reported in different studies when organic TM was supplemented to the broiler diets [2, 9, 23]. However, other researchers have reported variable responses with the addition of organic TM on growth performance. Zhu et al. [6] also reported that there was no negative impact on growth performance due to the supplementation of the diet with organic TM at reduced levels (30% and 50% of the regular inclusion level). Similar results were also observed by De Marco et al. [12], who found that using organic TM sources (metal chelates of glycine and hydrate) in broiler diet could reduce the supplementation of Zn, Fe, Mn and Cu to 50% of the strain recommendations. On the other hand, Sirri et al. [24] reported that substitution of Zn, Mn, and Cu from inorganic sources by their organic chelates (chelated metals methionine hydroxy analogue) significantly improved body weight gain and FCR of broilers from 0 to 51 days of age. The previous study in Vanaraja chickens also demonstrated the positive effect of CTM-supplemented diets, even at low dietary inclusion level (50%), on FCR during the entire experimental period [25]. The inconsistencies in the efficacy of organic minerals may be attributable to different factors. The breed, differences in diet composition, housing condition, type and dosage of the supplement, the duration of the experiment, and bird characteristics can affect the growth response of broiler chickens to organic mineral supplements. In previuos studies, dietary supplementation of organic acids is also reported to decrease intestinal pathogenic bacteria [26], improve gut morphology [27], and promote digestive enzyme activity and intestinal mucosal barrier function [28]. Therefore, it hypostasized that an increase in the absorptive surface area of the small intestine, as well as the development of beneficial bacteria in the gut, by organic acid supplementation may result in improvements in the digestibility and absorption of nutrients, which in turn may improve growth performance. However, an important part of the explanation behind the improved growth performance observed in broiler chickens receiving the organic acid-chelated TM in this study can be due to the advanced chelated structure of this supplement. Although organic acids seems to marginally promote growth performance, the novel and important point is that the spatial structure of these molecules in the coordination sphere of metal ions provides the best conditions for chelating minerals, and hence, plays an important role in the appearance of biological properties.

In this study, in addition to growth parameters, the evaluations of mineral digestibility and tibia characteristics were within the main purposes of this study. When minerals are added beyond the requirement of the animals, more is excreted because of the reduction in utilization efficiency for that mineral [9]. Decreasing the use of TM in poultry feeds may be a feasible solution for minimizing mineral emissions from poultry farms. In the present study, diet CTM25 could efficiently improve P, Zn, Mn, and Cu digestibility as compared to inorganic mineral supplementation. In addition, as feeding the diet supplemented with 50% of organic TM produced the higher Zn and Mn absorption than the use of 100% of inorganic forms in the current study, it can be said that providing 50% of organic TM levels could lead to higher mineral retention. Several researchers have stated the low excretion of organic TM through the excreta and consequently their high retention rate in broilers [6, 12], laying hens [29] and growing ostriches [15]. Based on the current results, it appears that lower supplementation of TM to 50% of the commercial level could improve the absorption of TM, without compromising the growth performance of broilers as previously mentioned. Increased mineral digestibility in broilers fed the diets supplemented with chelated TM is attributed to the fact that organic minerals may be better absorbed as they are more likely to be stable throughout the gastro-intestinal tract and less prone to antagonisms and interactions with other components of the digesta [11]. A possible reason for higher digestibility of P in the CTM25 and CTM 50 groups is that chelated form of TM may reduce the formation of complexes between P and other metal ions, and, therefore, increase P absorption in the ileum. Another possible reason to explain the greater AID of P might be the improvement in phytate P utilization due to organic acid released from CTM supplement [15], although the mechanism of action is not well-understood.

Trace minerals appear to play important roles in the growth, development, and maintenance of normal bone. Bone as a complex heterogeneous tissue is responsible for supporting muscle, and therefore there is a close link between growth and development of bone with overall body growth [30]. Tibia morphological measurements, such as tibia length, weight, ash, and mineral contents have been used as indicators for the evaluation of bone status in poultry [2]. Among the experimental group, the NTM group had the lowest values of tibia length and tibia ash, Zn, Mn, and Cu contents, which suggests that a lower amount of bone components was available in group NTM. In the present study, tibia morphological traits and bone mineral content in the CTM50 group were in the same range as those obtained by chickens in the ITM100 group, which could be due to greater mineral absorption and bioavailability in the CTM50 group. These results are in agreement with the results of previous studies [9, 13, 25], in which a stimulatory effect of organic mineral sources on morphological parameters of bone were observed. The same results were also found by M’Sadeq et al. [2], who indicated that broiler chickens fed 37.5 and 50% of organic yeast proteinate TM premix had no effect on tibia strength, weight, length, and width compared with those fed 75% inorganic TM premix or 100% salt encrusted TM premix on day 38 of age. Increased tibia P content in broilers fed the CTM50 and CTM100 diets compared to those fed the ITM diet in the present study also confirms the hypothesis that organic TM supplementation could reduce interference from agents that form insoluble complexes with ionic TM [25].

The metabolomic analysis of serum in the present study revealed that experimental groups only differed in uric acid concentration, which was higher in the CTM100 group than the NTM and ITM groups, while it was not different from the CTM25 and CTM50 groups. The increase in blood uric acid concentration could be associated with the free radical scavenging capacity and recognised as an important antioxidant in birds [31]. In a previous study where broiler chickens were fed diets that replaced inorganic TM with organic TM, the authors found a significantly higher serum uric acid level [1]. In part, these findings could contribute to the possible reduction of tissue damage by organically complexed minerals.

Malondialdehyde (MDA) is the principal product of lipid peroxidation, and its accumulation can reflect the degree of lipid peroxidation [32]. In contrast, the enzymatic scavengers, such as GSH-Px, SOD, and CAT enzymes, help break up the damage process by removing reactive oxygen species [33]. Therefore, SOD, GSH-Px, CAT, and MDA are often used as effective indicators to objectively reflect the antioxidant status in animals [34, 35]. As the antioxidant status of chickens was assessed, all TM-supplemented groups, except CTM25 group, exhibited an increase in serum GSH-Px and SOD activities when compared to the NTM group, while the CAT activity remained relatively constant in these chickens (Table 7). Feeding diets with CTM at the 25 and 50% of dose level suggested by the strain recommendations (CTM25 and CTM50) had the same activities of GSH-Px and SOD enzymes, as well as the same MDA level, as those observed in broilers fed diets containing sufficient amounts of inorganic TM (ITM group). This may indicate that organic minerals supplemented at lower levels have higher bioavailability and can exert the same antioxidant capacity than higher levels of inorganic forms. Similarly, in another study, total replacement of high levels of inorganic TM by lower levels (50% and 62.5% of the commercial recommended levels) of organic TM in broiler breeder diets had similar activities of serum GSH-Px and SOD [4]. Additionally, we found that birds fed the CTM100 diet had the highest GSH-Px activity in serum among all of the experimental groups in the present study. In a previous study, replacing inorganic TM (Fe, Mn, Zn, and Cu from sulfates) by 70% the levels of organic TM (complexed glycinates) improved hepatic antioxidant status in commercial broiler breeder hens [36]. The antioxidant stress protection was stated to be effective only if there are adequate quantities of cofactors such as Zn, Mn, Cu, and Se available [15, 37]. Organic Zn, Mn, and Cu have been reported to increase the synthesis of SOD [14, 38], while organic Se previously enhanced GSH-Px activity [39, 40], which is similar to our study. The use of organic TM might have increased the bioavailability of these minerals and thus reduced the accumulation of reactive oxygen species, which might have improved the antioxidant defense system via increasing GSH-Px activity in broilers receiving the CTM100 diet, as compared with those fed the ITM diet in this study.

## Conclusions

The present study indicated that in a typical corn-soybean diet, no TM supplementation adversely influenced broiler growth performance, bone mineralization, and antioxidant status. Our results also confirmed that supplemental ITM (a combination of Fe, Zn, Mn, Cu, Se, I, and Cr) was effectively replaced with reduced levels of CTM (25 and 50% of commercial levels) by maintaining broiler performance, bone mineralization, and antioxidant status, indicating that advanced chelate technology can decrease TM requirements of broiler chickens. Under commercial conditions, using CTM totally replacing inorganic TM could also improve performance index and antioxidant enzyme activity. However, further investigations are still required to elucidate the detailed mechanisms of the beneficial effects of this advanced technology based supplement on bird health and productivity.

## Data Availability

The datasets analysed in the present study are available from the corresponding author upon request.

## Abbreviations

ADFI: Average daily feed intake
ADG: Average daily gain
BW: Body weight
Ca: Calcium
CAT: Catalase
Cu: Copper
FCR: Feed conversion ratio
CTM: Chelated trace minerals
Cr: Chromium
EPI: European performance index
GIT: Gastrointestinal tract
Fe: Iron
GSH-Px: Glutathione peroxidase
I: Iodine
K: Potassium
MDA: Malondialdehyde
Mg: Magnesium
Mn: Manganese
Na: Sodium
P: Phosphorus
Se: Selenium
SOD: Superoxide dismutase
TAC: Total antioxidant capacity
TM: Trace minerals
Zn: Zinc

## References

[1] Echeverry H, Yitbarek A, Munyaka P, Alizadeh M, Cleaver A, Camelo-Jaimes G (2016). Organic trace mineral supplementation enhances local and systemic innate immune responses and modulates oxidative stress in broiler chickens. Poult Sci.

[2] M’Sadeq SA, Wu SB, Choct M, Swick RA (2018). Influence of trace mineral sources on broiler performance, lymphoid organ weights, apparent digestibility, and bone mineralization. Poult Sci.

[3] Sun J, Liu D, Shi R (2015). Supplemental dietary iron glycine modifies growth, immune function, and antioxidant enzyme activities in broiler chickens. Livest Sci.

[4] Wang G, Liu LJ, Tao WJ, Xiao ZP, Pei X, Liu BJ (2019). Effects of replacing inorganic trace minerals with organic trace minerals on the production performance, blood profiles, and antioxidant status of broiler breeders. Poult Sci.

[5] Underwood EJ, Suttle NF (1999). The mineral nutrition of livestock.

[6] Zhu Z, Yan L, Hu S, An S, Lv Z, Wang Z (2019). Effects of the different levels of dietary trace elements from organic or inorganic sources on growth performance, carcass traits, meat quality, and faecal mineral excretion of broilers. Arch Anim Nutr.

[7] Nollet L, Huyghebaert G, Spring P (2008). Effect of different levels of dietary organic (bioplex) trace minerals on live performance of broiler chickens by growth phases. J Appl Poult Res.

[8] Ramos-Vidales D, Gómez-Verduzco G, Cortes-Cuevas A, del Río-García JC, Fernández-Tinoco S, Chárraga-Aguilar S (2019). Organic trace minerals on productive performance, egg quality and immune response in Bovans White laying hens. J Anim Physiol Anim Nutr (Berl).

[9] Singh AK, Ghosh TK, Haldar S (2015). Effects of methionine chelate- or yeast proteinate-based supplement of copper, iron, manganese and zinc on broiler growth performance, their distribution in the tibia and excretion into the environment. Biol Trace Elem Res.

[10] Seyfori H, Ghasemi HA, Hajkhodadadi I, Hafizi M (2019). Effects of water supplementation of an organic acid-trace mineral complex on production and slaughter parameters, intestinal histomorphology, and macronutrient digestibility in growing ostriches. Poult Sci.

[11] Bao YM, Choct M (2009). Trace mineral nutrition for broiler chickens and prospects of application of organically complexed trace minerals: a review. Anim Prod Sci.

[12] De Marco M, Zoon MV, Margetyal C, Picart C, Ionescu C (2017). Dietary administration of glycine complexed trace minerals can improve performance and slaughter yield in broilers and reduces mineral excretion. Anim Feed Sci Technol.

[13] Kwiecień M, Winiarska-Mieczan A, Milczarek A, Tomaszewska E, Matras J (2016). Effects of zinc glycine chelate on growth performance, carcass characteristics, bone quality, and mineral content in bone of broiler chicken. Livest Sci.

[14] Aksu DS, Aksu T, Özsoy B, Baytok E (2010). The Effects of replacing inorganic with a lower level of organically complexed minerals (Cu, Zn and Mn) in broiler diets on lipid peroxidation and antioxidant defense systems. Asian Australas J Anim Sci.

[15] Seyfori H, Ghasemi HA, Hajkhodadadi I, Nazaran MH, Hafizi M (2018). Growth performance, mineral digestibility, and blood characteristics of ostriches receiving drinking water supplemented with varying levels of chelated trace mineral complex. Biol Trace Elem Res.

[16] Hafizi M, Hajarizadeh A, Atashi A, Kalanaky S, Fakharzadeh S, Masoumi Z (2015). Nanochelating based nanocomplex, GFc7, improves quality and quantity of human mesenchymal stem cells during in vitro expansion. Stem Cell Res Ther.

[17] Fakharzadeh S, Hafizi M, Baghaei MA, Etesami M, Khayamzadeh M, Kalanaky S (2020). Using nanochelating technology for biofortification and yield increase in rice. Sci Rep.

[18] Mohammadi V, Ghazanfari S, Mohammadi-Sangcheshmeh A, Nazaran MH (2015). Comparative effects of zinc-nano complexes, zinc-sulphate and zinc-methionine on performance in broiler chickens. Br Poult Sci.

[19] Aviagen. Ross 308 broiler. In: Nutrition Specification [RB Limited, editor]. Newbridge, Midlothian: Aviagen; 2014.

[20] Ghasemi HA, Nari N (2020). Effect of supplementary betaine on growth performance, blood biochemical profile, and immune response in heat-stressed broilers fed different dietary protein levels. J Appl Poult Res.

[21] AOAC. International Official methods of analysis of AOAC. International 18th ed. Arlington: Association of Analytical Chemists; 2006.

[22] Short FJ, Gorton P, Wiseman J, Boorman KN (1996). Determination of titanium dioxide added as an inert marker in chicken digestibility studies. Anim Feed Sci Technol.

[23] Olukosi OA, Van Kuijk S, Han Y (2018). Copper and zinc sources and levels of zinc inclusion influence growth performance, tissue trace mineral content, and carcass yield of broiler chickens. Poult Sci.

[24] Sirri F, Maiorano G, Tavaniello S, Chen J, Petracci M, Meluzzi A (2016). Effect of different levels of dietary zinc, manganese, and copper from organic or inorganic sources on performance, bacterial chondronecrosis, intramuscular collagen characteristics, and occurrence of meat quality defects of broiler chickens. Poult Sci.

[25] Rao SVR, Prakash B, Kumari K, Raju MVLN, Panda AK (2013). Effect of supplementing different concentrations of organic trace minerals on performance, antioxidant activity, and bone mineralization in Vanaraja chickens developed for free range farming. Trop Anim Health Prod.

[26] Dittoe DK, Ricke SC, Kiess AS (2018). Organic acids and potential for modifying the avian gastrointestinal tract and reducing pathogens and disease. Front Vet Sci.

[27] Nari N, Ghasemi HA, Hajkhodadadi I, Farahani AHK (2020). Intestinal microbial ecology, immune response, stress indicators, and gut morphology of male broiler chickens fed low-phosphorus diets supplemented with phytase, butyric acid, or *Saccharomyces boulardii*. Livest Sci.

[28] Yang X, Liu Y, Yan F, Yang C, Yang X (2019). Effects of encapsulated organic acids and essential oils on intestinal barrier, microbial count, and bacterial metabolites in broiler chickens. Poult Sci.

[29] Yenice E, Mızrak C, Gültekin M, Atik Z, Tunca M (2015). Effects of organic and inorganic forms of manganese, zinc, copper, and chromium on bioavailability of these minerals and calcium in late-phase laying hens. Biol Trace Elem Res.

[30] Loveridge N (1993). Micronutrients and longitudinal growth. Proc Nutr Soc.

[31] Simoyi MF, Van Dyke K, Klandorf H (2002). Manipulation of plasma uric acid in broiler chicks and its effect on leukocyte oxidative activity. Am J Physiol Regul Integr Comp Physiol.

[32] Tan L, Rong D, Yang Y, Zhang B (2019). The effect of oxidized fish oils on growth performance, oxidative status, and intestinal barrier function in broiler chickens. J Appl Poult Res.

[33] Zhu W, Chen S, Li Z, Zhao X, Li W, Sun Y (2014). Effects and mechanisms of resveratrol on the amelioration of oxidative stress and hepatic steatosis in KKAy mice. Nutr Metab.

[34] Akhavan-Salamat H, Ghasemi HA (2019). Effect of different sources and contents of zinc on growth performance, carcass characteristics, humoral immunity and antioxidant status of broiler chickens exposed to high environmental temperatures. Livest Sci.

[35] Ismail M, Al-Naqeeb G, Mamat WAAB, Ahmad Z (2010). Gamma-oryzanol rich fraction regulates the expression of antioxidant and oxidative stress related genes in stressed rat’s liver. Nutr Metab.

[36] Umar Yaqoob M, Wang G, Sun W, Pei X, Liu L, Tao W (2020). Effects of inorganic trace minerals replaced by complexed glycinates on reproductive performance, blood profiles, and antioxidant status in broiler breeders. Poult Sci.

[37] Jing CL, Dong XF, Wang ZM, Liu S, Tong JM (2015). Comparative study of DL-selenomethionine vs sodium selenite and seleno-yeast on antioxidant activity and selenium status in laying hens. Poult Sci.

[38] Jankowski J, Ognik K, Kozłowski K, Stępniowska A, Zduńczyk Z (2019). Effect of different levels and sources of dietary copper, zinc and manganese on the performance and immune and redox status of turkeys. Animals.

[39] Boostani A, Sadeghi AA, Mousavi SN, Chamani M, Kashan N (2015). Effects of organic, inorganic, and nano-Se on growth performance, antioxidant capacity, cellular and humoral immune responses in broiler chickens exposed to oxidative stress. Livest Sci.

[40] Dalia AM, Loh TC, Sazili AQ, Jahromi MF, Samsudin AA (2017). The effect of dietary bacterial organic selenium on growth performance, antioxidant capacity, and Selenoproteins gene expression in broiler chickens. BMC Vet Res.

